# Health promotion via SMS improves hypertension knowledge for deaf South Africans

**DOI:** 10.1186/s12889-017-4619-7

**Published:** 2017-08-18

**Authors:** Hanne Jensen Haricharan, Marion Heap, Damian Hacking, Yan Kwan Lau

**Affiliations:** Health and Human Rights Programme, School of Public Health and Family Medicine, Health Science Faculty, University of Cape Town Anzio Rd, Observatory, Cape Town, 7925 South Africa

**Keywords:** SMS, Cell phones, Text messages, mHealth, Health promotion, Health information, Health literacy, Healthy behaviour, Deaf, South Africa

## Abstract

**Background:**

Signing Deaf South Africans have limited access to health information. As a result, their knowledge about health is limited. Cell phone usage in South Africa is high. This study aimed to assess whether a short message service (SMS)-based health promotion campaign could improve Deaf people’s knowledge of hypertension and healthy living. Additionally, the study aimed to assess the acceptability of using SMSs for health promotion targeting Deaf people.

**Methods:**

A baseline questionnaire assessed participants’ knowledge about hypertension before an SMS-based information campaign was conducted. After the campaign, an exit questionnaire was conducted, containing the same questions as the baseline questionnaire with additional questions about general acceptability and communication preferences. Results were compared between baseline and exit, using McNemar’s test, paired t-test and Wilcoxon signed-rank test. Focus groups aimed to get further information on the impact and acceptability of SMSs. The focus groups were analysed using inductive thematic analysis.

**Results:**

The campaign recruited 82 participants for the baseline survey, but due to significant loss-to-follow-up and exclusions only 41 participants were included in the analysis of the survey. The majority (60%) were men. Eighty percent were employed, while 98% had not finished high school. The campaign showed a statistically significant improvement in overall knowledge about hypertension and healthy living amongst participants. Six individual questions out of 19 also showed a statistically significant improvement. Despite this, participants in focus groups found the medical terminology difficult to understand. Several ways of improving SMS campaigns for the Deaf were identified. These included using using pictures, using ‘signed’ SMSs, combining SMSs with signed drama and linking SMS-campaigns to an interactive communication service that would enable the Deaf to pose questions for clarification. Focus groups suggested that participants who were hypertensive during the campaign adopted a healthier lifestyle.

**Conclusion:**

SMSs were effective in improving Deaf people’s knowledge of hypertension and healthy living. However, SMS-campaigns should be cognizant of Deaf people’s unique needs and communication preference and explore how to accommodate these.

**Trial registration:**

The research was registered with the Pan African Clinical Trial Registry on December 1, 2015. Identification number: PACTR201512001353476.

**Electronic supplementary material:**

The online version of this article (doi:10.1186/s12889-017-4619-7) contains supplementary material, which is available to authorized users.

## Background

The aim of this paper is to assess whether health information disseminated by SMSs can improve signing Deaf South Africans’ knowledge of hypertension and healthy living. Further, it aims to assess the acceptability of using SMSs for health promotion targeting Deaf people. In this paper, Deaf (capitalised) refers to those permanently, sensorily disabled people with congenital or early onset deafness and whose first language is South African Sign Language (SASL).

It is estimated that between 500,000 and 1,5 million South Africans are Deaf and communicate via South African Sign Language (SASL) [1]. Communication barriers in health care are a serious impediment for the Deaf, affecting their access to health care [2–6]. Studies show that Deaf signers have poorer health than the general population and are often mis-diagnosed and receive inadequate treatment for chronic conditions [5, 7]. A South African study showed that poor communication between Deaf South Africans and their health care providers resulted in delayed diagnosis, misdiagnosis and problems with adherence [3].

There is limited knowledge on Deaf people’s health knowledge. A study with American Deaf patients found their health literacy to be low [7], while Australian studies suggest that they lack access to preventative health care information and have limited health awareness [4, 8]. Two studies found that Deaf Americans’ knowledge of cardiovascular disease was lower than the hearing population [9, 10]. One of these studies found that only 49% of Deaf adults in the US could list chest pain as a symptom associated with a heart attack. In comparison, 90% of the general adult population linked chest pain with a heart attack [10]. No study on Deaf South Africans’ health knowledge was identified, but their literacy level is known to be low [11]. The average literacy skills of Deaf South African school leavers are at a level of a grade 4 learner [12]. Deaf people are likely to miss out on orally based communication such as radio and TV in addition to much written information, which is not designed to take account of their low literacy level. One study concluded that most written materials use a language level, which is much higher than Deaf people’s literacy level [13]. While oral and written communication is generally not accessible for Deaf people, communicating in sign language also pose problems. Because sign language is a language of so-called ‘limited diffusion’, it has very limited vocabulary in specialist areas such as health [14]. An illustrative example of this problem is mentioned in a study in the U.K., which found that there was no term for cholesterol [15]. Moreover, considering that much health information in South Africa is conveyed through health talks at the clinics without the presence of a sign language interpreter, Deaf patients are likely to miss out on this communication.

While conveying health information to Deaf people is a challenge, there are examples of successful health campaigns. Notably, several studies using videos with Deaf in the United States to create cancer awareness have been successful in improving short term knowledge [16–19]. However, a study on British Deaf people at high risk of cardio vascular disease, found that health promotion following their assessment did not result in a decrease in their risk [15]. In South Africa, health information for Deaf people is mainly carried out through Deaf organisations. Deaf South Africans can generally not afford internet [20].

The proliferation of cell phones has resulted in 90% of the population in developing countries having a cell phone [21]. In South Africa, the number of cell phones are higher than that of the population. The use of cell phones in health care has increased in recent years in the developing world [22]. Cell phones are used as adherence support and in behavior change and health information campaigns [23–30]. A systematic review found that SMSs had the potential to support health behaviour changes in the short-term [31]. Further, a recent systematic review of reviews [21] found that SMSs were effective in addressing behavior change and adherence. A randomized control trial in Latin America found that mHealth interventions resulted in a small reduction in bodyweight and improvements in dietary habits amongst prehypertensive [32]. However, an evaluation of an SMS-campaign with hypertensive patients in Cape Town, South Africa, found no statistically significant improvement in health knowledge, though self-reported behaviour changed [33]. Studies on the usage of SMSs in health care amongst the Deaf are lacking. Through our previous work with Deaf South Africans, we knew that the use of cell phones was widespread amongst the Deaf. Cell phones have subsequently been identified as the preferred source of communication [20]. However, despite cell phones being used to send and receive messages amongst the Deaf, health interventions using cell phones are lacking.

Hypertension is one of many chronic diseases that are on the rise in the developing world, including South Africa. In South Africa, the prevalence is estimated to be around 21% [34]. No prevalence study on hypertension amongst the Deaf was identified, but there is no reason to believe that it should be lower than the general population.

We hypothesized that an SMS-based health information campaign could improve knowledge of hypertension and healthy living amongst the Deaf participants. We based the hypothesis on the low health literacy level and the widespread use of cell phones amongst the Deaf. The hypothesis was also based on an assumption that the prevalence of hypertension in the Deaf population would be similar to the general South African population. In addition, we took the results of a similar study with hypertensive hearing patients into consideration. This study did not result in a statistically significant improvement in knowledge. However, the study with hearing patients had a different starting point. The hearing hypertensive patients’ knowledge scores at baseline were high, leaving little room for measuring improvement. We assumed – based on our long-term association with the Deaf and international literature on Deaf health literacy [4, 7–10] – that this was not the case for our target group.

Our study is based on an understanding that access to information is both a human right central to the right to health and a prerequisite for healthy choices that can be protective against chronic diseases such as high blood pressure. The notion of ‘informational access’ is outlined in the General Comment 14 on the Covenant of Economic, Social and Cultural Rights [35], which South Africa has ratified. Both the Health Belief Model [36] and the Stages of Change Model [37] recognise knowledge as an important step in choosing healthy behaviour. Thus a campaign that aims at improving informational access can be viewed as a first step in improving health and the right to health.

## Methods

The study aimed to explore whether a SMS-based health information campaign for Deaf people can improve their knowledge about hypertension and healthy living. Further, the study aimed to assess the acceptability of using a SMS campaign for health promotion targeting Deaf people.

### Study population

The study population was Deaf people in Cape Town over the age of 18 years, who communicate via South African Sign Language (SASL).

### Study design

The study was designed as a pre-test-post-test study. While a controlled trial could have provided stronger evidence, this was not feasible. Deaf people in Cape Town are part of a very close community and the chance that participants would share information was deemed to be high. This would render a controlled trial invalid. Data collection took place in 2013–2014. Data analysis took place in 2014–2015.

The South African Deaf population poses a challenge for recruitment and sampling as they are not registered in any database. The group is geographically dispersed and use a wide variety of health services. To access Deaf people, this project relied on a long term association with Deaf people and their organisations such as the Deaf Community of Cape Town (DCCT) to recruit participants. Our study population was a convenience sample. Potential participants were invited to an information meeting where the research was explained in sign language. Further, a DVD explained the project and the consent process in sign language. Participants were invited to enrol in the programme on a separate occasion. We aimed to recruit 50 participants. This was based on the number of people attending the information meetings and reactions to the research at this meeting. We had approximately 100 people attending the information meetings. We were conservative in our estimation because this study was the first of its kind and we were unsure of the Deaf population’s response. We ended up recruiting 82 participants, but unfortunately had a high loss-to-follow up (28), and had to exclude another 13 participants for various reasons. This resulted in only 41 participants being included in the analysis.

The SMS-campaign (see Appendix 1) with information on hypertension and tips on healthy living was based on a similar SMS-campaign with hearing hypertensive patients, which was designed in collaboration with local health promoters [33]. It contained 20 SMSs with medical information on hypertension such as symptoms and possible consequences of hypertension and 57 SMSs with information on how high blood pressure could be avoided or managed through healthy living such as eating habits and exercise. Four SMSs explained the campaign and the option to opting out of the campaign. The campaign was piloted with Deaf research assistants and necessary amendments made. All research instruments were translated from English into Afrikaans and isiXhosa, the other two written languages used by the Deaf in the greater Cape Town area. The 81 SMSs were distributed over 28 weeks.

Prior to the SMS-campaign, participants completed a baseline questionnaire (see Appendix 2), containing 19 questions gauging their knowledge of hypertension and healthy living. Fifteen questions had simple binary answers, while four had multiple correct answers. An exit questionnaire (see Appendix 3) was administered shortly after the completion of the campaign. The exit questionnaire contained the same questions as the baseline, but with eight additional questions about communication preferences and acceptability of SMSs. These questions had either binary answers or multiple answers.

After analysis of the quantitative data, two focus groups were conducted with people who had received the majority of SMSs. The research team monitored the delivery of SMSs. People had the option of ‘opting out’ of the campaign. Further, SMS delivery failed if people changed phone numbers, left them uncharged or switched off for a period of time. Other reasons for delivery failure was people sharing phones with friends or family, having more than one phone and frequent change of phone numbers. Some people lost their phones during the campaign. To the focus groups, we invited only those individuals that we knew had received more than 80% of our SMSs. The focus groups were conducted after the survey had been analysed. An interview guide was developed aiming at exploring the quantitative results, the impact of the campaign and the acceptability of the SMSs. The focus groups were conducted by one researcher via a sign language interpreter. The focus groups (the interpreter’s voice over) were recoded via a dictaphone and transcribed.

To determine whether there was a change in knowledge between baseline and exit, each question was analysed as well as a total score. Each question either had a single correct answer or multiple correct answers. The single answer data were coded into binary outcomes with “1” signifying correct answer and “0” signifying incorrect answers. Correct and incorrect answers were then compared between baseline and exit using McNemar’s test. For the multiple answer data, the score for each individual question was tallied. Correct answers were worth “1”, incorrect “-1”. “Don’t know” was worth “0”. Because of the relatively small sample size, a Wilcoxon signed-rank test was used for all multiple answer data.

The overall score was calculated by tallying the scores of each individual question for each participant at both baseline and exit. Since the difference between the mean scores was not normally distributed, a Wilcoxon signed-rank test was conducted. For the additional questions about communication preferences and acceptability of the SMSs asked at exit, for both the binary and multiple answer questions, the proportion of respondents indicating each choice is reported. The qualitative data was analysed by the lead author using inductive thematic analysis.

### Ethics, consent and permission

Written project information sheets and consent forms were made available in the languages used in Cape Town: English, isiXhosa and Afrikaans. Research assistants who were proficient in SASL took signed consent and ensured that consent was informed and voluntary. The study was approved by the University of Cape Town’s Health Science Faculty Human Research Ethics Committee (nr. 043/2011).

## Results

The campaign recruited 82 participants, but only 41 participants were retained for the analysis due to loss-to-follow up and exclusions of people who had not received/were uncertain about receiving the SMSs or people who had not filled out the baseline questionnaire. See Additional file 1 for recruitment data.

The mean age of the participants that were included in the analysis was 45 years. More men (60%) than women participated in the survey. The majority, 50%, were married, and the majority were employed (80%). It is worth noting that the low educational level - 98% had not finished high school - is not unusual for Deaf South Africans. No data was available on how many participants were hypertensive. For a summary of the demographic profile of participants, see Table 1.

**Table 1:** Demographics

Demographics for participants included in the survey data analysis	
Demographic	n (%)
Total sample (N)	41
Mean age in years (SD)	45 (12)
Gender	
Male	25 (60)
Female	18 (40)
Marital status	
Divorced	3 (8)
Married	21 (50)
Single, living alone	2 (5)
Single, living with family	7 (17)
Single, living with partner	7 (17)
Widow	1 (3)
Education level	
Below Grade 7/Standard 5	16 (39)
Between Grade 7/Standard 5 and Grade 12	24 (59)
Passed Grade 12/Matric	0 (0)
Have post-matric qualification	1 (2)
Employment status	
Employed	33 (80)
Pensioner	3 (8)
Unemployed	5 (12)
Monthly income	
None	9 (22)
Pension/social grant	5 (12)
Less than R4000	19 (46)
Between R4000 and R10000	8 (20)
More than R10000	0 (0)
Preferred language for reading and writing	
Afrikaans	7 (17)
English	31(76)
isiXhosa	3 (7)

An analysis of participants’ knowledge before and after the SMS-campaign showed a significant increase in the overall test score between baseline and follow up (*p* = .0033). Table 2, below, shows the﻿ difference in knowledge for questions with one correct answer.

**Table 2:** Number of people with adequate knowledge at baseline and exit (n=41)

Question	Baseline	Exit	P-value
1: What is high blood pressure?	8 (19.51%)	18 (43.90%)	0.0213
2: What is normal blood pressure?	11 (26.83%)	15 (36.59%)	0.4240
3: Does everybody who has high blood pressure have symptoms?	18 (43.90%)	24 (58.54%)	0.2379
4: Should a person with high blood pressure stop taking his/her medication without asking the doctor?	33 (80.49%)	35 (85.37%)	0.7744
5: Can high blood pressure be cured?	16 (39.02%)	29 (70.73%)	0.0072
6: If a person with high blood pressure leaves Cape Town for a while, which one of the following is right?	28 (68.29%)	33 (80.49%)	0.0213
7. Is smoking good or bad if you have high blood pressure	37 (90.24%)	36 (87.80%)	
8. Can a person lower his/her blood pressure?	27 (65.85%)	37 (90.24%)	0.0213
9. Is keeping a normal weight important if you have high blood pressure?	26 (63.41%)	29 (70.73%)	0.6291
10. How much alcohol should you drink, if at all, if you have high blood pressure?	18 (43.90%)	22 (53.66%)	0.4545
11. How long should a person with high blood pressure exercise on at least three days of the week?	9 (21.95%)	15 (35.59%)	0.1796
12. Do eating habits affect blood pressure?	23 (56.10%)	26 (63.41%)	0.6636
13. Is salt good or bad for a person with high blood pressure?	17 (41.46%)	17 (41.46%)	
14. Are vegetables and fruit good for a person with high blood pressure?	36 (87.80%)	39 (95.12%)	0.4531
15. What should a person cut down on if you have high blood pressure?	22 (53.66%)	30 (73.17%)	0.0455

Table 3, below, shows the knowledge scores for questions with multiple correct answers.

**Table 3:** Knowledge scores for questions with multiple correct answers (n=41)

Question	Test	P-value
1.What puts a person at risk of developing high blood pressure?	Paired t test	0.0017
2. What complications can occur if a person has high blood pressure and does not control it?	Wilcoxon signed rank test	0.1570
3. A person with high blood pressure should go to the clinic if he/she has which of the following symptoms?	Wilcoxon signed rank test	0.0514
4. What are the best ways to manage stress?	Paired t test	0.7812

Six out of the 19 questions showed a statistically significant increase when analysed individually. In addition, the overall score showed a significant increase. The overall increase and the increase for each of the six questions are reflected in Table 4, below.

**Table 4:** Summary of questions with significant increase as well as overall increase

Question	Test	P-Value
1. What is high blood pressure?	McNemar’s	.0213
3. What puts a person at risk of developing high blood pressure?	Paired t test	.0017
7. Can high blood pressure be cured?	McNemar’s	.0072
8. If a person with high blood pressure leaves Cape Town for a while, which one of the following is right?	McNemar’s	.0213
10. Can a person lower his/her blood pressure?	McNemar’s	.0213
18. What should you cut down on if you have high blood pressure? Choose as many answers as you like.*	Wilcoxon signed-rank	.0455
OVERALL SCORES	Wilcoxon signed-rank	.0033

Most participants thought that the SMSs were effective in improving their knowledge of hypertension (80%). The majority of participants (78%) found the SMSs easy to understand, though 13% stated that they were not.

To a question about what participants liked or disliked about the campaign, 89% stated that they liked the SMSs because it gave them information, while 71% found them trustworthy. For 61% of the participants, the SMSs made them feel cared for by the health facilities, while 54% stated that the SMSs were entertaining. None of the respondents indicated that they did not like the SMSs.

SMSs were chosen by most people as the source of information they normally get information from (46%). This was followed by written material (24%), internet (17%), friends, family and colleagues (15%). Notably, 20% of the participants stated that they do not receive any health information. However, more than half (56%) stated that they felt that Deaf organisations were best at conveying health information; while 27% considered SMSs best. For a summary of results on acceptability and communication preference, see Table 5.

**Table 5:** Acceptability of SMSs amongst participants in hypertension survey

Acceptability of SMSs among participants who completed the hypertension survey	
Evaluation of SMS campaign	n (%)
Total sample (N)	41
Read all or most of the SMSs about hypertension	41 (100)
Felt that the SMSs improved hypertension knowledge	33 (80)
Felt that the SMSs were easy to understand	32 (78)
Found the SMSs useful	40 (98)
Found the SMSs informative	36 (89)
Found the SMSs trustworthy	29 (71)
Felt that somebody cared about me/us	25 (61)
Found the SMSs entertaining	22 (54)
Found the information irrelevant	3 (7)
Found the SMSs to be irritating	3 (7)
Did not like the SMSs	0 (0)
Normally get their health information from SMSs	19 (46)
Felt SMSs were the best way of giving information to Deaf people	11 (27)

### Qualitative data

The two focus groups had a total of 26 participants. The demographics of the focus group participants was largely similar to those included in the survey analysis, though their mean age was 2 years higher. They also had a slightly higher educational level, with only 15% having below grade 7 compared to 39% of the participants included in the survey analysis. Nine (1/3 of focus group participants) indicated that they suffered from hypertension during the campaign. The focus groups confirmed the health knowledge gap amongst the participants. Constantly, participants would interject to get information, seek clarification or ask specific health related questions. The SMS-campaign was seen as a way of addressing the knowledge gap. In particular, focus groups confirmed that messages about healthy living, diet and exercise had an impact on participants. Overwhelmingly, participants expressed satisfaction with the campaign. Some participants argued that they ‘understood everything’ or ‘understood clearly’ and appreciated the ‘simple language’ and the briefness of the SMSs. There was agreement that the SMS-campaign improved their knowledge. The quote below is an example of this:

*When I received the SMSs I was wow! I was very interested when I received the SMSs. I was happy because I never got any teachings from elsewhere so I learned more and more from the SMSs.* Participant 1.

Most participants liked that the information was repeated, and many indicated that they kept the SMSs on their phone so they could keep the information and use the SMSs to remind them later. One participant commented that he wrote the SMSs in a notebook. However, there were also participants who argued that they had difficulties with understanding the SMSs. In particular, they struggled with medical terminology. The difficulties resulted in many questions for clarification during the focus groups. The quote below is on example of how participants struggled to understand the meaning of the SMS text.

*The reason why I struggled to understand the words. If you could just make it shorter so that I can understand because I don’t know much about English. Uhm… there are words that I actually ( …) I don’t actually understand what does it mean.* Participant 2

Another participant who struggled with understanding the SMSs suggested that “the medical term should be changed with a synonym that’s easy to understand”. Many participants linked their difficulty in understanding some of the information to their poor educational background. While participants saw SMSs as an important way of conveying information, they also stressed that the information conveyed by SMSs was not always sufficiently detailed. Some explained that they would take the SMSs to family or relatives to get more information. Many suggested that SMSs should be combined with an interactive communication service, where participants could write back and ask for explanations or further information. The quote below is an illustration of this:

*If I receive a SMS and the word is so difficult for me; can I sent you back a SMS saying that I don’t understand this word. Is that possible?* Participant 4.

Suggestions were also made on how to make SMSs easier to understand. These included using simpler language or using ‘sign language structure’, referring to the unique grammar and word order of sign language. The use of pictures in SMSs was also advocated by participants who explained that Deaf people are more familiar with visual communication.

*I think if you could put a picture maybe to explain a difficult word. Like put a picture of the fruit and then write the name of the fruit as well. That’s just an idea because it’s easy for Deaf people to identify a word by seeing a picture. If you put a picture then you put a word that means what that picture is. So that would be another easy thing to do.* Participant 5

However, others felt that it was important that SMSs were in plain English and introduced medical terminology as this would improve their medical knowledge and be useful in the clinical setting. Signed video messages were another suggestion on how to improve the campaign, as suggested below:

*All in all I think in the future you can just video instead of sending SMSs. You can take a camera and video someone signing the whole thing instead of SMSing.* Participant 3.

While many participants agreed that signed video clips would improve understanding, concerns were raised whether the type of phones most Deaf South Africans use are technologically advanced enough for video messaging. There were also many suggestions made about using visual forms of communication such as posters and drama, which is used by Deaf organisations to convey health information. In particular, drama was seen to be a very effective way of communicating about health issues. Currently, however, health dramas for Deaf South Africans are mainly about HIV, and participants said they would appreciate health drama about other health issues. There was consensus that combining methods would be preferable, as indicated below. The quote below refers both to combining information sources and the importance of using words (rather than just sign).

*I will say DCCT has the best way of showing to Deaf people using drama…. But still I would prefer UCT (University of Cape Town) because they are introducing us to words so when you go to the hospital then you know there was a word on your SMSs and then it’s familiar. So I would suggest both of them. Because UCT brings words to us. Please they have to be a combination.* Participant 3

While we did not set out to explore the impact of the campaign on health seeking behaviour, the focus groups demonstrated the potential of this. Some participants reported that they decided to have their hypertension checked as a result of receiving information, as shown in the quote below:

Being Deaf, I don’t know much about hypertension. The SMSs made me go to the clinic to get a check-up. Usually [I] don’t go to the clinic because there is no interpreter. Participant 4.

In other instances, the information in the SMSs was used to care for others, as the following SMS indicates.

*My mom has high blood pressure so I showed her [the SMSs] and she found it very useful. I feel good about the SMSs as I didn’t have any information before. They were not giving enough information to her (the participant’s mom) at the clinic. I am now taking care of her – for example cooking and making her exercise a bit. The SMSs were very good and had an impact.* Participant 5

Several participants said that they shared the information with friends and family like one participant who explained that he had become a platform to inform people about health and kept the SMSs on his phone so he could show them to people who had little knowledge of hypertension. Another participant explained that friends had their blood pressure checked after she shared the SMSs with them.

*Friends don’t know about hypertension, they think I make it up. I showed them that the SMSs came from UCT to convince them. They went to the doctor for a check-up after learning from the SMSs. They want information too now.* Participant 6

While we had not targeted hypertensive participants, 1/3 or the focus groups, i.e. nine individuals, reported to be suffering from hypertension. These participants reported that the SMSs impacted on their health seeking behaviour. They said they had changed eating habits, begun to exercise and attend clinic regularly. The quote below illustrates this:

*I loved eating braaied (barbequed) meat, oh I loved it. I loved eating cake, chips and all that junk food. But then I received the SMSs from UCT. They taught me, showed me what was the right food to eat and what was not good for me to eat, so now I stopped eating too much cake. Now I eat just a little… I have changed the way that I’m eating. Also due to the junk that I ate I experienced a lot of sickness and high blood (colloquial term for hypertension), but after leaving that junk food because of the SMSs my health became normal. I’m healthy now.* Participant 7.

## Discussion

The findings showed that SMSs can improve Deaf people’s knowledge about hypertension and healthy living. We have not identified any other study that showed that SMS-campaigns are effective in improving health knowledge of the Deaf. A similar campaign with hearing hypertensive South Africans [33], did not show a statistically significant improvement in health knowledge. This may suggest that SMSs may be particularly effective for the Deaf. A reason could be that Deaf participants’ knowledge was lower than hearing participants’ at baseline and the potential for impact therefore greater. Comparing these two studies confirms that Deaf South Africans health literacy about hypertension is lower than that of the hearing population. This is in line with studies on Deaf health literacy in general [4, 7, 8] and hypertension [9, 10].

While the study overall showed a statistically significant increase in knowledge, it is worth noting that only six out of 19 questions contained in the study’s questionnaire showed statistically significant increase individually. Nine additional questions showed improvement, but this was not statistically significant. One question showed no change, while another question showed a decrease in knowledge. Further studies should explore and evaluate the questions with limited or no knowledge improvement in light of comments about language and medical terminology made in focus groups. This could be used to improve the campaign and also highlights the importance of piloting any campaign before it is rolled out on a bigger scale.

The focus groups highlighted a huge health literacy gap amongst Deaf South Africans, and the study showed that SMSs are an effective and acceptable way of improving their health knowledge. The impact of the SMS-campaign is likely to be influenced by the fact that many participants found the SMSs trustworthy and that the SMSs made them feel cared for. However, the focus groups also suggested that the information delivered through SMSs was perceived to be insufficient and that understanding was sometimes compromised. The focus groups highlighted that despite overall improvement in knowledge, some participants found the medical terminology challenging. This is not surprising given that several studies argue that Deaf people’s health literacy level in general and on cardiovascular diseases in particular is low [4, 7–10]. In order to improve accessibility, future campaigns would have to consider how to address this by paying close attention to language level and the use of medical terminology. Deaf focus group participants’ suggestion that words should be explained is worth pondering over. However, in the SMS-campaign reported on here, we wanted to keep SMSs to 150 characters in order to capture one message in one SMS and thus there was limited space for explaining concepts. Other suggestions worth exploring would be to adapt the written language and use pictures, as suggested by some focus group participants.

A possible way of improving the SMS - campaign suggested in the focus groups should be given some consideration. This is the proposal that future campaigns should use ‘signed’ SMSs as small video clips which convey the content of the SMSs in sign language, which is then sent at regular intervals similar to the written SMSs. This suggestion should be seen in the light of Deaf people’s low written language literacy level and their preference for sign language. It is evident that signed SMSs may be a way of avoiding the problems encountered by written messages because of participants low written language literacy levels. Another potential advantage of a signed SMS-campaign could be that it would be easier to convey more detailed information, requested in the focus groups. In addition, it is possible that it would be easier to explain medical terminology. While signed SMSs are promising, it is imperative to explore whether the phones used by the majority of Deaf South Africans have the technical capacity to receive these. It would also be important to consider the cost of data for a signed campaign.

When considering the advantages of signed SMSs it is worth noting that sign language lacks vocabulary in many specialist areas, including health [14]. In Australia, a Medical Signbank is being developed to improve signs related to health and medicine [14]. Until a similar initiative is taken with regards to SASL, SMSs and other health communication using sign will have to rely on explaining medical terms in sign. Ideally, developing a signbank simultaneously with developing health awareness campaigns delivered via sign for the Deaf should be considered.

In considering the best mode of communicating health issues to the Deaf, one should also pay attention to the view of some focus group participants, who argued that it was important that the SMSs contained medical terms as it introduced them to written medical terminology. They found that being exposed to medical terms helped them in health care consultations. In that way, the written SMSs helped bridge the gap between the Deaf participants and the medical world. Given that most medical consultations involving Deaf South Africans occur without a sign language interpreter, it can be valuable to improve Deaf people’s knowledge of written medical terminology, which they may encounter in medical consultations, where the Deaf often rely on written notes between themselves and the health care provider [3]. 

We did not identify other SMS-based health promotion campaigns, but there are other health information campaigns specifically designed for the Deaf. The authors of a non-systematic review [13] notes internet training workshops, peer education programs and four video-based campaigns directed at increasing Deaf Americans’ cancer awareness [16–19]. All four studies on videos found that they improved health awareness and thus it is worth comparing video-based and SMS-based campaigns for the Deaf. It can be argued that there are pros and cons of both and that context should be given consideration. The obvious advantage of videos compared to a written SMS-campaign is that they can use sign language and avoid some of the problems posed by written language use. In that regard, they are similar to a signed SMS-campaign. However, getting people together for screening of a video could be challenging in a country such as South Africa as it will exclude people in more remote areas, posing a geographical limit to access. Further, the four studies on using video assessed impact two months after the showing of the video and long-term knowledge retention is unknown as it is in our study. In this regard, it is interesting to reflect on some of the comments made by participants in our focus groups, namely that they kept the SMSs on their phone to be able to look at them at a later stage and that they liked the fact that information was repeated. Both signed and written SMSs can be stored on mobile phones and easily retrieved. This is not possible for a video that was screened as a once of event. In some of the studies videos were uploaded to the internet and made accessible to anyone with access to the internet. However, in a South African context, the majority of Deaf have limited internet access because they cannot afford internet or do not have the necessary computer literacy skills [20].

Another important implication, which should be studied further, is how the SMS-campaign empowered Deaf people to seek more information. SMSs became part of a learning and information-seeking process where participants were motivated to seek more information, even though it is clear that they have limited options. Friends and family often had to assist with more information. For some, the SMSs empowered them in making decisions regarding their health. They received information on high blood pressure for the first time and acted on it (self-reported), for instance by having their blood pressure measured. The SMSs also empowered participants as patients as they used their newly gained information in medical consultations. For those that were hypertensive, it resulted in self-reported behaviour change with regards to diet and exercise. Conceptualising SMSs as part of a learning process means seeing them as a tool to empower Deaf people as patients and bridge the gap between their ‘signing’ world and the medical world of words.

Interestingly, focus groups suggested that this was a collective learning. Deaf South Africans tend like other Deaf populations to function as a distinct social group and findings suggest that they shared the SMSs. There were also examples where knowledge was used to inspire others to adopt more healthy behaviour, including seeking medical help. The impact of such campaigns may therefore expand beyond the study population to the wider Deaf community and help this particular population become more empowered patients.

An important finding, which further research should focus on, is the potential impact of SMS-campaigns on health seeking behaviour. Those participants who were hypertensive indicated that the information prompted them to adopt a healthier lifestyle. As this behaviour change is self-reported, it is difficult to assess whether it reflects actual behaviour change or social desirability. However, it should at least be seen as an indication of willingness to adopt a healthy lifestyle or a first step in the process. It is worth noting that only nine participants were hypertensive and further research is necessary to determine the impact of SMS-campaigns on behaviour change amongst the general and hypertensive Deaf population. These findings are consistent with studies, which found SMSs to have a short term impact on health seeking behaviour amongst hearing people with chronic diseases [21, 31, 32]. These studies called for longitudinal studies to assess long term impact. This call should be extended to hard-to-reach populations such as the Deaf. The potential of information campaigns such as the one reported on here to impact on behaviour change can be justified with reference to the Health Belief Model and the Stages of Change Model, which theorise that improved information is the first step in initiating changes in health seeking behaviour [36, 37].

The results from the focus groups point to the importance of taking cognizance of the needs of a specific target group when designing health information campaigns. Deaf people have special needs and a preference for visual communication such as pictures, posters and drama. They may prefer ‘video signed’ messages. Further studies should explore how to combine different information sources and modes of communication. Deaf campaigns should also explore using both sign and written text. While written SMSs have the advantage of introducing Deaf people to written terminology, which can be useful in medical consultations, signs may be better at improving knowledge. Furthermore, Deaf communication campaigns should consider how they can include interactive forms of communication that would enable Deaf participants to ask questions and seek further information and clarification. This would afford Deaf people the opportunity to not only be passive recipients of information, but also be able to actively engage with that information and address an issue raised repeatedly in the focus groups: the need to seek more detailed information and clarity. Interestingly, a South African health app for Deaf, which is currently being conceptualised, is ﻿considering combining text and sign [20].

The study also highlights the importance of taking context into consideration. While videos may be an effective way of communicating with the American Deaf population, it may be problematic in a South African context, where internet is not economically accessible for the majority of Deaf people. Combining SMSs with videos and internet resources could be promising, but currently its value may be limited in the South African context.

Randomized controlled studies with Deaf populations would provide stronger evidence for the effectiveness of SMS-campaigns. Further research focusing on Deaf hypertensive patients would be useful to assess the effect on this target group. Such studies should aim both at improving knowledge and impact on health-seeking behaviour such as diet and exercise. Similar campaigns with other chronic diseases may be equally interesting.

### Strengths and limitations

The paper is to the best of our knowledge the first that explores the use of SMSs to convey knowledge to improve health for Deaf people. It contributes to studies that explore ways of addressing Deaf people’s limited health knowledge and resulting health disparities. The relative small sample size as well as loss-to-follow should be noted as limitations. Further, it should be noted that the study was done in a metropolitan area and used convenience sampling. This may limit the generalizability of the study. It is very likely that Deaf people residing in more rural areas have a lower literacy level than those residing in urban areas. It is possible that those who decided to take part in the study had a particular interest in the topic or in health information in general. It is equally conceivable that loss-to-follow up could partly be a result of people who chose not to complete the study because the found the SMSs difficult to understand. Thus selection-bias could be an issue. Lack of data on how many survey participants were hypertensive presents another limitation. A further limitation is that there may be volunteer bias amongst those attending the focus groups. Their educational level was slightly higher. Those limitations notwithstanding, the study presents promising results that should be explored further.

## Conclusion

This study demonstrates, to our knowledge for the first time, that SMSs are effective in improving Deaf people’s knowledge of hypertension and healthy living. It also showed an SMS-campaign to be acceptable to the target population. The results of this study should be explored further. The impact of SMS-campaigns on health seeking behaviour is promising, but should be researched further.

## Appendix 1

**ᅟ:** SMS campaign

English SMSs: Hypertension Deaf-campaign
Welcome to SMSs about high blood pressure.
Greetings: You can know the SMSs are from us, they will start with ‘health tips’ and ‘did you know’. We will use BP as short for ‘blood pressure’.
We know that you may not have high BP. But it is still good to learn about the condition and about healthy living.
Health tip: Let’s get started. A good way to lower BP is to exercise. Walking, exercising at home or going to a sports club helps.
Did you know? Blood pressure is the force/pressure of blood flowing through the arteries in your body.
Health tip: BP can become lower. Quitting smoking is one of the changes that can help.
Did you know? Blood pressure more than 130/80, puts a person at risk of developing hypertension (high BP). Higher than 130/80 is high BP.
Health tip: Knowing your BP is important. Ask your nurse and doctor at your next visit to the clinic.
Health tip: High BP can be lowered by eating less salt and red meat, including sausages.
Did you know? Healthy eating helps control high blood pressure.
Health tip: A person with high BP should maintain a healthy weight and lose weight if he/she is overweight.
Did you know? High BP cannot be cured. But a healthy way of life can help manage it.
Did you know? By changing lifestyle, a person with high BP can manage his/her condition.
Health tip: Healthy lifestyle changes for people with high BP: Eat more fish, skinless chicken & vegetables. Eat very little red meat & very little salt.
Did you know: High BP is a chronic disease. It means a person will have it always. But he/she does not have to feel ill.
Health tip: Stress can cause high BP. A person with high BP should try to find ways to de-stress.
Health tip: Good ways to lower stress are exercising or talking about problems. By managing stress, a person can lower his/her BP.
Did you know? Smoking, drinking, unhealthy eating habits, being overweight, too little exercise and stress put you at risk of high BP.
Did you know? Changing your way of life helps to lower blood pressure. If you need more information, speak to doctor or nurse.
Health tip: Eating less fat from animals helps to lower BP. One way to do this is to cut fat off meat before cooking it.
Did you know? A person with high BP, should contact a doctor/clinic if they have symptoms, such as pain in chest, stomach cramps or persistent headaches.
Health tip: A person with high BP should try to cut down on red meat and salt.
Did you know? A person may have no symptoms and still have high blood pressure. BP should be checked regularly.
Did you know? No symptoms, does not mean that you do not have high blood pressure. A person with high BP should visit the clinic regularly.
Health tip: Remember that exercising can help control high BP.
Health tip: A person with high BP should NEVER change his/her medication or stop taking it unless a doctor tells them to.
Health tip: A person with high BP should go to the clinic to get medication for the time they will be away when they leave Cape Town.
Health tip: A person with high BP should never share medication with friends and family.
Health tip: High BP can be lowered by eating very little salt. One way of doing this is NOT to add salt to the food before tasting.
Did you know? A person with high BP should not stop taking medication because they feel better. You can have high BP without having symptoms.
Did you know? A person with high BP should see his/her doctor regularly to manage their blood pressure.
Did you know? If you do not want to continue receiving SMSs, please send a “please call me” to 076280 8585.
Health tip: Good ways to manage high BP: manage your stress, lose weight if you are overweight, change eating habits.
Did you know? If you have high BP, you need to see a doctor regularly to manage your condition. A person with high BP should not miss medical check-ups.
Health tip: Tips on how to lower BP, if you have high BP: manage your stress, lose weight if you are overweight, change eating habits.
Did you know? Keeping a healthy weight is important if you have high blood pressure.
Health tip: There are many things a person with high BP can do to lower it. One way is to lose weight if overweight.
Health tip: Managing BP can prevent complications such as a stroke, heart attack or kidney damage.
Health tip: High BP can be managed by taking medicine, seeing a health care provider regularly, and changing way of life.
Health tip: Other tips on lowering BP: Stop smoking and drinking alcohol, eat healthily, exercise, maintain healthy weight, and do not stress too much.
Health tip: A person with high BP must contact a doctor if they have chest pain, stomach cramps, lots of headaches, shortness of breath, swollen legs.
Did you know? A person with high BP can lower risk of illness by taking medicine, attending clinic appointments, changing way of life.
Health tip: Exercise and changing eating ways can help a person with high BP lose weight and lower BP.
Health tip: Remember high BP can be controlled by: Exercise, stop smoking, manage stress, lose weight or change what you eat.
Health tip: Changing the way you live can help control high blood pressure. Many small changes help.
Health tip: Exercise helps control BP & keep a healthy weight. A person with high BP can try fast walking, climbing stairs, dancing, & exercise at home
Health tip: Aim at exercising 30 min at least three times a week - this is good for people with high BP.
Health tip: Alcohol can increase blood pressure. Stop drinking or reduce your drinking to lower your BP if you have high BP.
Health tip: If you are a woman, you should not drink more than one alcoholic drink per day. If you are a man, two alcoholic drinks is the limit.
Health tip: Remember one drink means one beer OR one tot of spirit OR one glass of wine OR one small glass of sherry.
Health tip: Remember you can only have one alcoholic drink a day if you are a woman, two drinks if you are a man.
Health tip: Want better health and lower high BP? Try this: Exercise, stop smoking, reduce or stop drinking alcohol.
Did you know? Smoking is bad if you have high blood pressure. Stop smoking to reduce BP if it is high.
Health tip: Eat less salt if you have high BP. One way of doing this is not to add salt to the food before tasting. Try it today.
Health tip: Stress can cause high blood pressure. Stress can be managed by exercising. The more the better!
Health tip: People with high BP can manage stress by exercising and talking about their problems.
Health tip: Healthy eating habits can lower high blood pressure for people with high BP. They also help people to lose weight & keep a healthy weight.
Health tip: People with high BP should eat more vegetables, fruit, beans and lentils. All these help to keep blood pressure low.
Health tip: Tips for people with high BP: Cook vegetables in little water with no salt or fat (butter, margarine or oil).
Health tip: Tips for people with high BP: Cut down on fat. Too much fat is bad for a person with high BP. Especially fat from animals (meat, milk, eggs).
Health tip: Remember for people with high BP, better to cook with oil or Flora/Rama margarine NO butter. Only little animal fat.
Did you know? If you do not want to continue receiving SMSs, please send a “please call me” to 076280 8585
Health tip: People with high BP need to cut down on fatty food and deep fried foods such as pies, sausages and chips.
Health tip: Fruit and vegetables are good if you have high BP. Try and eat 5 fruit and vegetables per day.
Health tip: Want to eat more vegetables? Try adding vegetables to rice, pasta, stews, curries. Vegetables are good for people with high BP.
Health tip: Feel like a snack? Try a raw carrot, or tomatoes or cucumbers or fruit. Much healthier than chips and cakes - for people with BP & others.
Health tip: Take-away food (like KFC) is often high in fat and salt. Better for people with BP & others to cook their own healthy meals.
Health tip: Tip for people with high BP: Use soft tub margarine instead of hard brick margarine.
Health tip: Boil, grill and steam rather than fry. It is a good way to cut down on fat for people with high BP.
Did you know? Reducing salt intake can reduce your BP. Remember, salt will raise your blood pressure.
Health tip: Cook with little salt and don’t add salt to your food if you have high BP. This can help lower high BP.
Health tip: Tip for people with high BP: Cut down on polony, salami, ham, bacon, sausages, dried and smoked fish. These have too much salt.
Health tip: People with high BP can lower their BP. They can have less salt intake by cutting down on salty snacks such as chips, peanuts and biscuits.
Health tip: Want to eat less fat? Eat more chicken without the skin and fish instead of red meat. Eating less fat is good for people with high BP
Health tip: Tip for people with high BP: Buy red meat without fat. Also cut all fat from meat to reduce the fat you eat.
Health tip: Tip for people with high BP: An easy way to reduce fat in the diet is to remove chicken skin before cooking. Most of the fat is in the skin.
Health tip: Choose low fat or skim milk and dairy products if you have high BP. This can help reduce high BP for people suffering from the condition.
Health tip: Small changes that help lower BP: Powder milk are high in fat. Better use low fat or skim milk.
Health tip: People with high BP can make small changes to lower their. BP. One way is to eat brown bread not white bread.
Health tips for lower BP are ending. You will not receive SMSs anymore. We hope you learned a lot about high blood pressure.

## Appendix 2

### Baseline questionnaire

1. **Reference__________**
2. **Consent signed?**

- Yes

3. **About yourself**

- What is your date of birth?
- **What is your reading /written language of choice?**
- English
- Afrikaans
- isiXhosa

4. **What is your marital status?**

- Married
- Single living with partner
- Single living with family
- Single living with people - not family
- Single living alone
- Widow/widower
- Divorced

5. **What is your highest level of schooling/education?**

- Below grade 7/standard 5.
- Between grade 7/standard 5, and 12/standard 10
- Passed Matric
- No matric but diploma
- Have post-matric qualification.
- Don’t know

6. **What is your employment status?**

- Employed
- Unemployed
- Pension
- Not looking for employment
- Never worked

7. **Your monthly income?**

- None
- Pension/social grant
- Less than R4000
- Between R4000 and R 10,000
- More than 10,000

8. **What is high blood pressure? Choose one answer.**

- Fat covering the arteries
- The force of blood flowing through the arteries
- Too many white blood cells
- Don’t know

9. **What is normal blood pressure? Choose one answer.**

- 120/80
- 130/80
- 90/70
- 140/100
- Don’t know

10. ** What puts a person at risk of developing high blood pressure? Choose as many answers as you like.**

- Smoking
- Drinking
- Unhealthy diet
- Too little exercise.
- Too much exercise.
- Eating too little
- Having unprotected sex.
- Stress.
- Being overweight.
- Being underweight.
- Too little sleep.
- Don’t know

11. ** Does everybody who has high blood pressure have symptoms? Choose one answer.**

- Yes, you will always have symptoms
- No, you can have no symptoms and still have high blood pressure
- Don’t know.

12. ** What complications can occur if a person has high blood pressure and does not control it? Choose as many answers as you like.**

- Stroke
- Cancer
- HIV
- Heart attack
- Epilepsy
- Kidney damage
- Diabetes
- Don’t know

13. ** Should a person with high blood pressure stop taking his/her medication without asking the doctor? Choose one answer.**

- Yes
- No
- Don’t know

14. ** Can high blood pressure be cured? Choose one answer.**

- Yes
- No
- Don’t know

15. ** If a person with high blood pressure leaves Cape Town for a while, which one of the following is right? Choose one answer.**

- He/she should stop taking his/her medication
- He/she should go to the clinic to get enough medication for the time he/she will be away
- Get medication from a clinic where he/she is going
- Don’t know

16. ** A person with high blood pressure should go to the clinic if he/she has which of the following symptoms? Choose as many answers as you like.**

- Persistent headache
- Feel nausea
- Chest pain
- Loose stools
- Stomach cramps
- Tiredness
- Ear ache
- Don’t know

17. ** Can a person lower his/her blood pressure? Choose one answer.**

- No there is nothing one can do
- Yes, changing one’s lifestyle helps control high blood pressure
- Don’t know

18. ** Is smoking good or bad if you have high blood pressure? Choose one answer.**

- Good
- Bad
- Neither good/nor bad
- Don’t know

19. ** Is keeping a normal weight important if you have high blood pressure? Choose one answer.**

- Yes
- No
- Weight does not matter as long as the person takes his/her medication
- Don’t know

20. ** How much alcohol should you drink, if at all, if you have high blood pressure? Choose one answer.**

- Men can drink 3 drinks daily, women 2
- As much as you like. It does not affect blood pressure
- Men can drink two, women one
- Don’t know.

21. ** How long should a person with high blood pressure exercise on at least three days of the week? Choose one answer.**

- 2 h
- It does not matter
- 30 min
- 1 h
- He/she should not exercise at all
- Don’t know

22. ** What are the best ways to manage stress? Choose as many answers as you like.**

- You can’t manage your stress
- Eat better
- Exercise
- Talk about your problems
- Drink more alcohol

23. ** Do eating habits affect blood pressure? Choose one answer.**

- No, not at all
- Yes, a healthy diet helps control high blood pressure
- Very little can be done to control high blood pressure
- Don’t know

24. ** Is salt good or bad for a person with high blood pressure? Choose one answer.**

- Too much salt is bad
- People with high blood pressure need more salt
- People with high blood pressure should have no salt
- Neither good/nor bad
- Don’t know

25. ** What should you cut down on if you have high blood pressure? Choose as many answers as you like.**

- Fish
- Red meat
- Chicken
- Vegetables
- Don’t know

26. ** Are vegetables and fruit good for a person with high blood pressure? Choose one answer.**

- Yes
- No
- Maybe
- Don’t know

## Appendix 3

### Exit Questionnaire

1. **Reference ________________**
2. **Consent signed?**
  - Yes
3. **Are you receiving medical treatment for high blood pressure or hypertension?**
  - Yes
  - No
4. **What is high blood pressure? Choose one answer.**
  - Fat covering the arteries
  - The force of blood flowing through the arteries
  - Too many white blood cells
  - Don’t know
5. **What is normal blood pressure? Choose one answer.**
  - 120/80
  - 130/80
  - 90/70
  - 140/100
  - Don’t know
6. **What puts a person at risk of developing high blood pressure? Choose as many answers as you like.**
  - Smoking
  - Drinking
  - Unhealthy diet
  - Too little exercise
  - Too much exercise
  - Eating too little
  - Having unprotected sex
  - Stress.
  - Being overweight.
  - Being underweight.
  - Too little sleep.
  - Don’t know
7. **Does everybody who has high blood pressure have symptoms? Choose one answer.**
  - Yes, you will always have symptoms
  - No, you can have no symptoms and still have high blood pressure
  - Don’t know
8. **What complications can occur if a person has high blood pressure and does not control it? Choose as many answers as you like.**
  - Stroke
  - Cancer
  - HIV
  - Heart attack
  - Epilepsy
  - Kidney damage
  - Diabetes
  - Don’t know
9. **Should a person with high blood pressure stop taking his/her medication without asking the doctor? Choose one answer.**
  - Yes
  - No
  - Don’t know
10. **Can high blood pressure be cured? Choose one answer.**
  - Yes
  - No
  - Don’t know
11. **If a person with high blood pressure leaves Cape Town for a while, which one of the following is right? Choose one answer.**
  - He/she should stop taking his/her medication
  - He/she should go to the clinic to get enough medication for the time he/she will be away
  - Get medication from a clinic where he/she is going
  - Don’t know
12. **A person with high blood pressure should go to the clinic if he/she has which of the following symptoms? Choose as many answers as you like.**
  - Persistent headache
  - Feel nausea
  - Chest pain
  - Loose stools
  - Stomach cramps
  - Tiredness
  - Ear ache
  - Don’t know
13. **Can a person lower his/her blood pressure? Choose one answer.**
  - No, there is nothing one can do
  - Yes, changing one’s lifestyle helps control high blood pressure
  - Don’t know
14. **Is smoking good or bad for a person with high blood pressure? Choose one answer.**
  - Good
  - Bad
  - Neither good/nor bad
  - Don’t know
15. **Is keeping a normal weight important for a person with high blood pressure? Choose one answer.**
  - Yes
  - No
  - Weight does not matter as long as the person takes his/her medication
  - Don’t know
16. **Should people with high blood pressure drink alcohol?**
  - Yes
  - No
  - Don’t know
17. **If a person with high blood pressure drinks alcohol, how many drinks can they have? Choose one answer.**
  - Men can drink 3 drinks daily, women 2
  - As much as you like. It does not affect blood pressure
  - Men can drink two, women one
  - Don’t know
18. **How long should a person with high blood pressure exercise on at least three days of the week? Choose one answer.**
  - 2 hours
  - It does not matter
  - 30 minutes
  - 1 hour
  - He/she should not exercise at all
  - Don’t know
19. **What are the best ways to manage stress? Choose as many answers as you like.**
  - You can’t manage your stress
  - Eat better
  - Exercise
  - Talk about your problems
  - Drink more alcohol
20. **Do eating habits affect blood pressure? Choose one answer.**
  - No, not at all
  - Yes, a healthy diet helps control high blood pressure
  - Very little can be done to control high blood pressure
  - Don’t know
21. **Is salt good or bad for a person with high blood pressure? Choose one answer.**
  - Too much salt is bad
  - People with high blood pressure need more salt
  - People with high blood pressure should have no salt
  - Neither good/nor bad
  - Don’t know
22. **What should a person with high blood pressure cut down on? Choose as many answers as you like.**
  - Fish
  - Red meat
  - Chicken
  - Vegetables
  - Don’t know
23. **Are vegetables and fruit good for a person with high blood pressure? Choose one answer.**
  - Yes
  - No
  - Maybe
  - Don’t know
24. **Have you received SMSs about hypertension? Please choose one answer.**
  - Yes
  - No
  - Not sure
25. **Did you read the SMSs about hypertension? Please choose one answer.**
  - Yes, I read all the messages
  - Yes, I read the messages most of the time
  - No, I did not read the message very often
  - No, I did not read the messages
26. **Did the SMSs improve your knowledge about high blood pressure?**
  - Yes
  - No
  - Not sure
27. **Were the SMSs easy to understand?**
  - Yes
  - No
  - Not sure
28. **Did you find the SMSs useful?**
  - Yes
  - No
  - Not sure
29. **Where do you normally get information about health?**
  - Written material
  - Internet
  - SMSs
  - Friends, family, colleagues
  - Other
  - I don’t get any information about health
30. **What do you think is the best way of giving information about health to Deaf people?**
  - Written material
  - Internet
  - SMSs
  - Friends, family, colleagues
  - DCCT and other organisations
  - Other
31. **What did you like/not like about the SMSs? Choose as many answers as you like.**
  - They were short and easy to understand
  - I found them trustworthy.
  - I found them irritating.
  - I found the information irrelevant
  - I found them entertaining
  - I felt that somebody cared about me/us
  - The gave me important information
  - I did not like the SMSs
  - Don’t know

## Additional file

Additional file 1: Participant diagram. (DOCX 27 kb)

## Abbreviations

BP: Blood pressure
DCCT: Deaf Confederation Cape Town
SMS: Short Message Service
UCT: University of Cape Town

## References

[1] Heap M, Morgans H. Language policy and SASL: interpreters in the public service. In: Watermeyer B, Swartz L, Lorenzo T, Schneider M, Priestly M (editors). Disability and social change: a South African agenda. Cape Town. South Africa. Human Sciences Research Council; 2006.

[2] Iezzoni LI, O’Day BL, Killeen M (2004). Communicating about health care: observations from persons who are deaf or hard of hearing. Ann Intern Med.

[3] Haricharan HJ, Heap M, Coomans F (2013). Can we talk about the right to healthcare without language? A critique of key international human rights law, drawing on the experiences of a deaf woman in Cape Town. South Africa. Disabil Soc..

[4] Napier J, Kidd MR (2013). English literacy as a barrier to health care information for deaf people who use Auslan. Aust Fam Physician.

[5] Emond A, Ridd M, Sutherland H, et al. The current health of the signing deaf community in the UK compared with the general population: a cross-sectional study. BMJ Open. 2015;5:e006668. doi:10.1136/bmjopen-2014-006668. 10.1136/bmjopen-2014-006668 PMC4316428 25619200

[6] Kritzinger J, Schneider M, Swartz L, et al. “I just answer ‘yes’ to everything they say”: access to health care for deaf people in Worcester, South Africa, and the politics of exclusion. Patient Educ Couns. 2014;94(3):379–83. doi:10.1016/j.pec.2013.12.006. Epub 2013 Dec 14. 10.1016/j.pec.2013.12.006 24388666

[7] Barnett S, McKee M, Smith SR (2011). Deaf sign language users, health inequities, and public health: opportunities for social justice. Prev Chronic Dis.

[8] Terry DR, Le Q, Nguyen HB. Moving forward with dignity: exploring health awareness in an isolated deaf community of Australia. Disabil Health J. 2016;9:281–8. http://dx.doi.org/10.1016/j/dhjo.2015.11.002. 10.1016/j.dhjo.2015.11.002 26905971

[9] McKee M, Schlehofer D, Cuculick J, et al. Perceptions of cardiovascular health in an underserved community of deaf adults using American sign language. Disabil Health J. 2011;4(3):192–7. doi:10.1016/j.dhjo.2011.04.001. 10.1016/j.dhjo.2011.04.001 PMC3378999 21723526

[10] Margellos-Anast H, Estarziau M, Kaufman G. Cardiovascular disease knowledge among culturally deaf patients in Chicago. Prev Med. 2006;42(3):235–9. doi:10.1016/j.ypmed.2005.12.012. 10.1016/j.ypmed.2005.12.012 16460789

[11] Glaser M, Lorenzo T. Developing literacy with deaf adults. In: Watermeyer B, Swartz L, Lorenzo T, Schneider M, Priestley M, (editors). Disability and social change: a south African agenda [internet]. Pretoria: Human Sciences Research Council; 2006 [cited 2015 May 18]. pp. 192–205.

[12] Aarons D, Akach P. South African sign language: one language or many? In: Mesthrie R (editor). Language in South Africa. Cambridge: Cambridge University Press; 2002.

[13] Kuenburg A, Fellinger P, Felling J (2016). Health care access among deaf people. J Deaf Stud Deaf Educ..

[14] Johnston T, Napier J (2010). Medical signbank. Bringing deaf people and linguists together in the process of language development. Sign Lang Stud.

[15] Patel JV, Gill PS, Chackathayil J, et al. Short-term effects of screening for cardiovascular risk in the deaf community: A pilot study. Cardiology Research Practice. 2011;493546. doi:10.4061/2011/493546. 10.4061/2011/493546 PMC3087949 21559268

[16] Kaskowitz SR, Nakaji MC, Clark KL (2006). Bringing prostate cancer education to deaf men. Cancer Detection and Prevention..

[17] Sacks L, Nakaji M, Harry KM (2013). Testicular cancer knowledge among deaf and hearing men. J Cancer Educ..

[18] Harry KM, Malcarne VL, Branz P (2012). Evaluating a skin cancer education program for the deaf community. J Cancer Educ.

[19] Choe S, Lim RS-H, Clark K (2009). The impact of cervical cancer education for deaf women using a video educational tool employing American sign language, open captioning, and graphics. J Cancer Educ.

[20] Chininthorn P, Glaser M, Tucker WD, et al. Exploration of deaf people’s health information sources and techniques for information delivery in Cape Town: a qualitative study for the design and development of a mobile health app. JMIR Hum Factors. 2016;3(2):e28. doi:10.2196/humanfactors.6653. 10.2196/humanfactors.6653 PMC5124113 27836819

[21] Hall A, Cole-Lewis H, Bernhardt JM. Mobile text messaging for health: a systematic review of reviews. Annu Rev Public Health. 2015;36:393–415. doi:10.1146/annurev-publchelath-031914-122855. 10.1146/annurev-publhealth-031914-122855 PMC4406229 25785892

[22] Deglise C, Suggs ML, Odermatt P (2012). SMS for disease control in developing countries: a systematic review of mobile health applications. J Telemed Telecare.

[23] Chen Z, Fang L, Chen L (2008). Comparison of an SMS text message and phone reminder to improve attendance at a health promotion center: a randomized controlled trial. J Zhejiang Univ Sci B.

[24] Patrick K, Griswold WG, Raab F (2008). Health and the mobile phone. Am J Prev Med.

[25] Lim M, Hocking J, Hellard M (2008). SMS STI: a review of the uses of mobile phone text messaging in sexual health. Int J STD AIDS.

[26] Alverson DC, Holtz B, D’lorio J et al. One size doesn’t fit all: bringing telehealth services to special populations. Telemed J E Health. 2008;14(9):957–63. doi:10.1089/tmj.2008.0115. 10.1089/tmj.2008.0115 19035807

[27] Doarn CR. Technology implications in global health. World Medical & Health Policy. 2010;2(1):Article 22. doi:10.2202/1948-4682.1064.

[28] Chan CV, Kaufman DR (2010). A technology selection framework for supporting delivery of patient orientated health interventions in developing countries. J Biomed Inform.

[29] Leon N, Surender R, Bodrow K, et al. Improving treatment adherence for blood pressure lowering via mobile phone SMS-messages in South Africa: a qualitative evaluation of the SMS-text adherence suppoRt (StAR) trial. BMC Fam Pract. 2015;16(1):80. doi:10.1186/s12875-015-0289-7. 10.1186/s12875-015-0289-7 PMC4490665 26137844

[30] Lau YK, Cassidy T, Hacking D, et al. Antenatal health promotion via short message service at a midwife obstetrics unit in South Africa: a mixed methods study. BMC Pregnancy Childbirth. 2014;14:284. doi:10.1186/1471-2393-14-284. 10.1186/1471-2393-14-284 PMC4158091 25145970

[31] Vodopivec-Jamsek V, de Jongh T, Gurol-Urganci I, et al. Mobile phone messaging for preventive health care. Cochrane Database Syst Rev. 2012;12:12:CD007457. doi:10.1002/14651858.CD007457.pub2. 10.1002/14651858.CD007457.pub2 PMC6486007 23235643

[32] Rubinstein D, Miranda JJ, Beratarrechea A, et al. Effectiveness of an mHealth intervention to improve the cardiometabolic profile of people with prehypertension in low-resource urban settings in Latin America: a randomised controlled trial. Lancet Diabetes Endocrinol. 2016;4(1):52–63. doi:10.1016/S2213-8587(15)00381-2. 10.1016/S2213-8587(15)00381-2 26653067

[33] Hacking D, Haricharan HJ, Brittain K, et al. Hypertension health promotion via short message service (SMS) at a community health centre in South Africa: a mixed method study. JMIR mHealth uHealth. 2016;4(1):e22. doi:10.2196/mhealth.4569. 10.2196/mhealth.4569 PMC4807246 26964505

[34] Steyn K. Hypertension in South Africa. In: Chronic diseases of lifestyle in South Africa since 1995–2005. Steyn K, Fourie J, Temple N (editors). Technical Report. Cape Town: South African Medical Research Council; 2006. pp. 80–96.

[35] UN Committee on Economic Social and Cultural Rights (CESCR). General Comment No 14: The Right to The Right to the Highest Attainable Standard of Health (Art 12 of the Covenant), 11 August, 2000, E/C. 12/2000/4, available at http://refworld.org/docid/4538838d0/html. [Accessed 1 Aug 2017].

[36] Ajzen I (2002). Perceived behavioural control, self-efficacy, locus of control and the theory of planned behaviour. J Appl Soi Psychol..

[37] Prochaska JO, DiClemente CC, Norcross JC (1992). In search of how people change: applications to addictive behaviours. Am Psychol.

