# Supplementary material for: Health promotion via SMS improves hypertension knowledge for deaf South Africans
Source: BMC Public Health. 2017 Aug 18;17:663. doi: 10.1186/s12889-017-4619-7 (PMC5563060; doi:10.1186/s12889-017-4619-7)
Supplement: Additional file 1: — Participant diagram. (DOCX 27 kb) [file 12889_2017_4619_MOESM1_ESM.docx]

Figure S1: Participant diagram flow diagram

Exclusions

13 were excluded because they

indicated that they:

1. had not received SMSs (n=10)
2. were unsure about receiving SMSs (n=3)
3. had not filled out the baseline survey (n=3)

Exclusions

Analysis:

41 participants were included in the analysis

28 participants were lost to follow-up

f follow-up

Follow-up:

54 participants completed the follow-up survey

Recruitment:

82 participants were recruited and completed the baseline survey
